# Supplementary material for: Splice-Junction-Based Mapping of Alternative Isoforms in the Human Proteome
Source: Cell Rep. Author manuscript; Available in PMC 2020 Jan 15. (PMC6961840; doi:10.1016/j.celrep.2019.11.026)
Supplement: 2 [file NIHMS1546469-supplement-2.zip › DF1/PXD010154/Heart/Heart_7_TTN_TPVQEEVIEVKVPEVPKKPVPEEK.pdf]

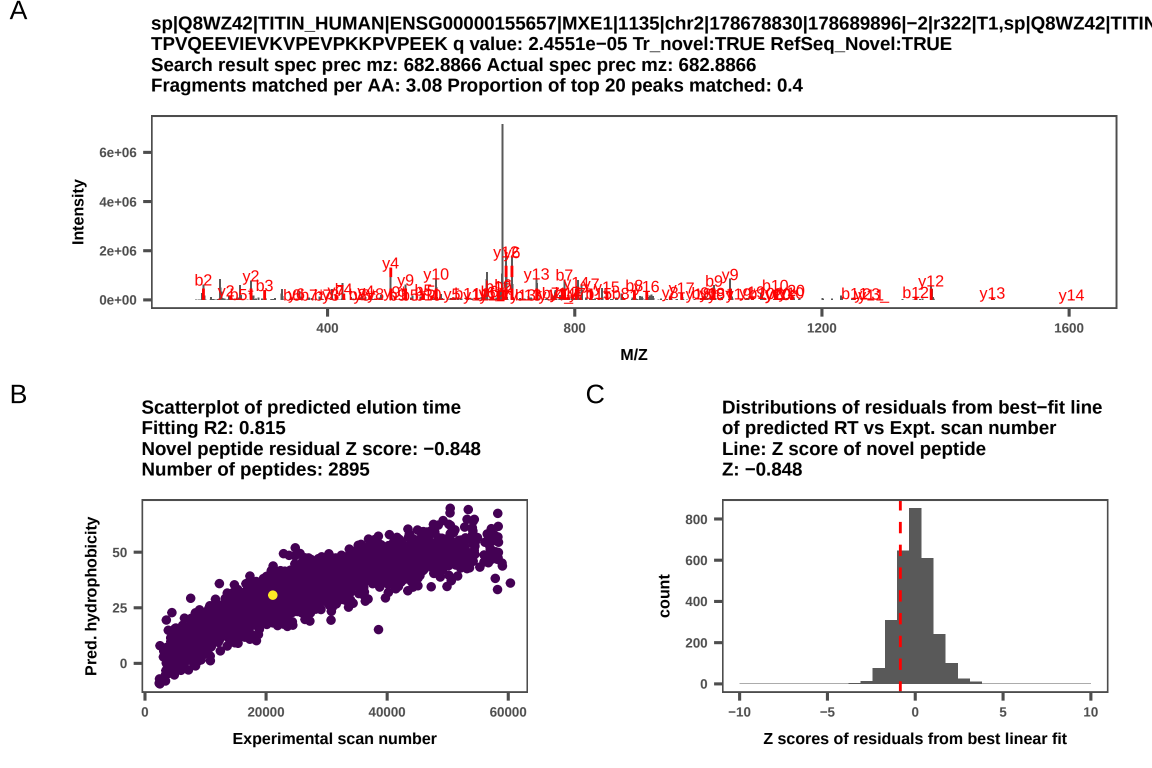

A
sp|Q8WZ42|TITIN_HUMAN|ENSG00000155657|MXE1|1135|chr2|178678830|178689896|−2|r322|T1,sp|Q8WZ42|TITIN
TPVQEEVIEVKVPEVPKKPVPEEK q value: 2.4551e−05 Tr_novel:TRUE RefSeq_Novel:TRUE
Search result spec prec mz: 682.8866 Actual spec prec mz: 682.8866
Fragments matched per AA: 3.08 Proportion of top 20 peaks matched: 0.4
Intensity
6e+06
4e+06
2e+06
0e+00
400
800
1200
1600
M/Z
B
Scatterplot of predicted elution time
Fitting R2: 0.815
Novel peptide residual Z score: −0.848
Number of peptides: 2895
Pred. hydrophobicity
50
25
0
0
20000
40000
60000
Experimental scan number
C
Distributions of residuals from best−fit line
of predicted RT vs Expt. scan number
Line: Z score of novel peptide
Z: −0.848
count
800
600
400
200
0
−10
−5
0
5
10
Z scores of residuals from best linear fit
